# Regulation of PKC-θ function by phosphorylation in T cell receptor signaling

**DOI:** 10.3389/fimmu.2012.00197

**Published:** 2012-07-11

**Authors:** Xiaohong Wang, Huai-Chia Chuang, Ju-Pi Li, Tse-Hua Tan

**Affiliations:** ^1^ Department of Pathology and Immunology, Baylor College of Medicine,Houston, TX, USA; ^2^ Immunology Research Center, National Health Research Institutes,Zhunan, Taiwan

**Keywords:** PKC-θ, phosphorylation, TCR signaling

## Abstract

Protein kinase C (PKC)-θ is a serine/threonine kinase belonging to the calcium-independent novel PKC subfamily; its expression is restricted to certain tissues and cell types, including T cells. The signals delivered from T cell receptor (TCR) and CD28 costimulatory molecules trigger PKC-θ catalytic activation and membrane translocation to the immunological synapse, leading to activation of NF-κB, AP-1, and NF-AT. These transcription factors are important for T cell survival, activation, and differentiation. Phosphorylation of PKC-θ at multiple Ser/Thr/Tyr residues is induced in T cells during TCR signaling. Some phosphorylation sites play critical roles in the regulation of PKC-θ function and downstream signaling. The regulation mechanisms for PKC-θ phosphorylation sites are now being revealed. In this review, we discuss the current understanding of the regulation of PKC-θ function by phosphorylation during TCR signaling.

## INTRODUCTION

Protein kinase C (PKC)-θ was originally cloned in 1993 as a novel member of the PKC gene family (Baier et al., 1993; Chang et al., 1993). Because PKC-θ is highly expressed in T cells, immunologists have examined the roles of PKC-θ in T cell activation and immune regulation. Nearly 20 years after its discovery, numerous studies using cell lines and genetic animal models demonstrate that PKC-θ plays essential roles in controlling peripheral T cell activation; preventing T cell anergy; regulating the development of regulatory T cells, T helper (TH)2, and TH17 cells; and modulating autoimmune pathogenesis (Marsland and Kopf, 2008; Zanin-Zhorov et al., 2011). The signals triggered by T cell receptor (TCR) and CD28 costimulatory molecules induce membrane translocation and kinase activation of PKC-θ, leading to subsequent activation of NF-κB and AP-1 (Baier-Bitterlich et al., 1996; Coudronniere et al., 2000; Sun et al., 2000; Bi et al., 2001). These transcription factors induce IL-2 expression and regulate T cell activation and functions. The regulatory mechanisms of the activation and functions of PKC-θ in T cells have been unraveled. Post-translational regulation of PKC-θ by phosphorylation plays important roles in regulating PKC-θ kinase activity and membrane translocation, both of which are essential for PKC-θ function in T cells. Here, we focus our discussion on the function and regulation of critical phosphorylation sites of PKC-θ in T cell activation. Identification of the important regulators of PKC-θ phosphorylation may provide novel therapeutic drug targets for autoimmunity.

## STRUCTURE BASIS FOR PKC-θ ACTIVATION

**FIGURE 1:**
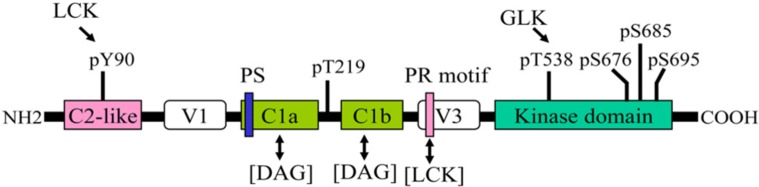
**Structural domains and phosphorylation sites of PKC-θ.** One-way arrow indicates the phosphorylation of the indicated site by a kinase. Two-way arrow indicates an interaction. PS, pseudosubstrate sequence; PR, proline-rich motif.

Human PKC-θ is composed of 706 amino acids. The basic structure of PKC-θ is shown in **Figure 1**. Like other PKC family members, PKC-θ consists of an N-terminal regulatory region (amino acids 1–378) and a C-terminal catalytic region (amino acids 379–706; Baier et al., 1993; Chang et al., 1993). The regulatory region displays the domain structure typical of PKC isoforms. The C2-like domain sequence is similar to the Ca^2+^-binding C2 domain sequences of other PKCs; however, it does not bind to Ca^2+^. The C2-like domain of PKC-θ contains a phosphorylated Y90 residue, which may mediate the interaction with an SH2 domain-containing protein, as seen in PKC-δ (Benes et al., 2005). In addition, the C2-like domain of PKC-θ may interact with a receptor for activated C kinase (RACK), which may regulate membrane translocation of PKC-θ (Schechtman and Mochly-Rosen, 2001). The interaction of a specific RACK with PKC-θ has not been reported; however, a PKC-δ/θ selective peptide blocking an RACK-binding site on PKC-δ/θ inhibits PKC-θ function (Nagy et al., 2009). Two tandem cysteine-rich C1 domains bind to diacylglycerol; the C1b domain has much higher affinity for diacylglycerol than the C1a domain (Melowic et al., 2007). A pseudosubstrate sequence (RRGAIKQA) within the C1a domain of PKC-θ binds to the substrate-binding region in the catalytic domain, inhibiting the PKC-θ kinase activity in the absence of allosteric effectors. C1 domains are flanked by two variable, or hinge, regions (V1 and V3). A recent study shows that the V3 domain is involved in the indirect association of PKC-θ with CD28, leading to PKC-θ membrane translocation into the immunological synapse; the interaction of lymphocyte-specific protein tyrosine kinase (LCK) with a proline-rich motif-like sequence in the V3 domain may be important for this action (Kong et al., 2011).

**FIGURE 2:**
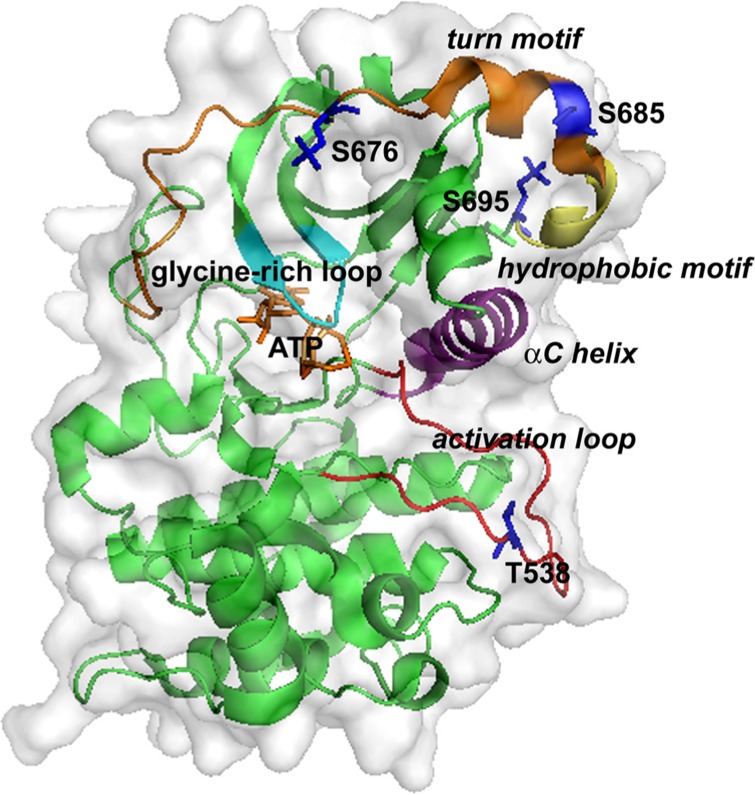
**Three-dimensional structure of the kinase domain of PKC-θ.** ATP binds at a cleft between the N-lobe (top) and C-lobe (bottom). Key structural elements are highlighted: the glycine-rich loop (cyan), the αC helix (purple), the activation loop (red), the turn motif (orange), and the hydrophobic motif (yellow). Phosphorylation sites and ATP are shown as sticks in blue and yellow-orange, respectively.

The crystal structure of the PKC-θ catalytic domain has been solved (Xu et al., 2004). The three-dimensional structure of PKC-θ is shown in **Figure 2**. The conserved core of the structure includes an N-terminal lobe and a C-terminal lobe, connected by a hinge linker sequence (Xu et al., 2004). The interface of the two lobes constitutes the active site cleft, which is responsible for the substrate binding and phosphate delivery (Xu et al., 2004). The key and conserved structural elements in the PKC-θ catalytic domain include a glycine-rich loop (GXGXXG) (involved in ATP binding and catalysis), an αC helix (participating in the substrate binding and catalysis), the activation loop bearing the essential phospho-threonine-538 (T538; critical for kinase activation), the hydrophobic motif containing phospho-serine 695 (S695), and the turn motif containing conserved phospho-serine 676 (S676) and phospho-serine 685 (S685) (Xu et al., 2004).

Similar to other protein kinases, PKC-θ displays two main conformational states: “open/active” and “closed/inactive” (Xu et al., 2004; Seco et al., 2012). In the inactive state, the pseudosubstrate sequence in the N-terminal regulatory region of PKC-θ forms intramolecular interaction with the substrate-binding region in the catalytic domain (House and Kemp, 1987). This prevents the catalytic domain from being accessible to substrates (House and Kemp, 1987). The allosteric change of PKC-θ from “closed” to “open” state involves two important mechanisms: diacylglycerol binding to the C1 domains and T538 phosphorylation at the activation loop (Seco et al., 2012). The signal transduction initiated from TCR/CD28 costimulation induces the generation of second messenger diacylglycerol, which binds to C1 domains, resulting in the exposure of the activation loop of PKC-θ (Melowic et al., 2007). The activation loop of PKC-θ is then accessible to phosphorylation by germinal center kinase-like kinase (GLK, also named MAP4K3) and subsequent catalytic activation (Chuang et al., 2011). Phorbol esters such as phorbol-12, 13-dibutyrate (PDBu) and phorbol myristate acetate (PMA) are potent non-physiological PKC-θ agonists that mimic the action of diacylglycerol and are widely used to induce PKC-θ activation (Ron and Kazanietz, 1999). Six phosphorylation sites have been identified on PKC-θ: Y90, T219, T538, S676, S685, and S695. These phosphorylation sites play distinct roles in the regulation of PKC-θ kinase activity or membrane translocation, which will be discussed below.

## FUNCTION AND REGULATION OF PKC-θ PHOSPHORYLATION SITES

### PKC-θ Y90 PHOSPHORYLATION

#### Identification and regulation of PKC-θ Y90 phosphorylation

PKC-θ is tyrosine phosphorylated in Jurkat T cells and primary T cells upon anti-CD3 with or without anti-CD28 costimulation (Liu et al., 2000; Bi et al., 2001). Coexpression with LCK, but not other tyrosine kinases including FYN, ZAP-70, SYK, or ITK, induces tyrosine phosphorylation of PKC-θ in COS-1 cells (Liu et al., 2000). Furthermore, tyrosine phosphorylation of PKC-θ is undetectable in LCK-deficient Jurkat T cells upon anti-CD3 stimulation (Liu et al., 2000). Additionally, LCK is constitutively and directly associated with PKC-θ in the transfected HEK293T cells and Jurkat T cells, and PKC-θ can be directly phosphorylated by LCK *in vitro* and *in vivo* (Liu et al., 2000). To identify the LCK-induced tyrosine phosphorylation site of PKC-θ, a series of PKC-θ-derived short peptides containing individual tyrosine residues are used as substrates for LCK in *in vitro* kinase assays (Liu et al., 2000). The peptide containing Y90, but not other tyrosine residues, is significantly phosphorylated by LCK *in vitro* (Liu et al., 2000). Furthermore, substitution of Y90 with phenylalanine abolishes LCK-induced PKC-θ tyrosine phosphorylation in transfected COS-1 cells and anti-CD3-stimulated Jurkat T cells (Liu et al., 2000). These data suggest that LCK directly phosphorylates PKC-θ at Y90. However, *in vivo* Y90 phosphorylation of PKC-θ during TCR signaling remains to be further demonstrated by immunoblotting using a phospho-specific antibody or confirmed by mass spectrometry.

#### Function of PKC-θ Y90 phosphorylation

Y90 phosphorylation of PKC-θ positively regulates NF-AT and NF-κB activation in T cells. The catalytically active PKC-θ A148E mutant induces NF-AT and NF-κB activation in Jurkat T cells, whereas the Y90F mutation of PKC-θ A148E mutant greatly reduces NF-AT and NF-κB activation (Liu et al., 2000; Bi et al., 2001). This suggests that Y90 phosphorylation regulates PKC-θ function, leading to downstream NF-AT and NF-κB activation. The evidence of LCK-induced Y90 phosphorylation and membrane translocation of PKC-θ suggests that Y90 phosphorylation may regulate the membrane translocation of PKC-θ (Liu et al., 2000; Bi et al., 2001). However, other studies suggest that LCK regulates membrane translocation via mediating the formation of PKC-θ/LCK/CD28 tri-partite interaction (Tavano et al., 2004; Hofinger and Sticht, 2005; Kong et al., 2011). In these studies, the SH3 domain of LCK interacts with the proline-rich motif in the V3 domain of PKC-θ, whereas the SH2 domain of LCK is bound to the phosphorylated Y207 of the CD28 cytoplasmic tail (Tavano et al., 2004; Hofinger and Sticht, 2005; Kong et al., 2011). PKC-θ mutations at the V3 proline-rich motif suppress membrane translocation of PKC-θ to the immunological synapse and also attenuate T cell activation upon anti-CD3/CD28 costimulation (Kong et al., 2011). It is unknown whether Y90 phosphorylation of PKC-θ is involved in the regulation of PKC-θ/LCK/CD28 tri-partite interaction. The role of Y90 phosphorylation in the regulation of PKC-θ membrane translocation needs to be demonstrated by more direct evidence, such as a study of membrane translocation of PKC-θ Y90F mutant in T cells during TCR signaling. In addition, whether Y90 phosphorylation regulates PKC-θ catalytic activity remains unknown.

### PKC-θ T219 Phosphorylation

#### Identification and regulation of PKC-θ T219 phosphorylation

T219 was originally identified as a major phosphorylation site of PKC-θ by phosphopeptide mapping (Thuille et al., 2005). The PKC-θ protein used for the phosphopeptide mapping is from purified baculovirus-expressed recombinant PKC-θ after *in vitro* kinase assays in the absence of exogenous substrates, suggesting that T219 is an autophosphorylation site of PKC-θ (Thuille et al., 2005). T219 phosphorylation of recombinant PKC-θ is further demonstrated by immunoblotting using a phospho-specific antibody, which does not recognize PKC-θ mutants with the T219 substitution with either alanine or glutamic acid (Thuille et al., 2005). In Jurkat T cells or primary T cells, PKC-θ T219 phosphorylation is induced by the phorbol ester PDBu, anti-CD3 alone, anti-CD3 plus anti-CD28, or vanadate (a tyrosine phosphatase inhibitor) (Thuille et al., 2005). The mechanism that induces T219 phosphorylation in TCR signaling remains unknown. However, T219 phosphorylation of PKC-θ is undetectable in the kinase-dead PKC-θ K409R mutant in Jurkat T cells upon cellular stimulation, suggesting that T219 is an inducible autophosphorylation site of PKC-θ during T cell activation (Thuille et al., 2005).

#### Function of PKC-θ T219 phosphorylation

T219 phosphorylation of PKC-θ is important for PKC-θ-mediated T cell activation upon TCR stimulation (Thuille et al., 2005). PKC-θ T219A mutant abrogates TCR-induced activation of NF-κB and NF-AT as well as subsequent IL-2 promoter transactivation in Jurkat T cells (Thuille et al., 2005). Interestingly, although loss of T219 phosphorylation impairs PKC-θ-mediated cellular function, PKC-θ T219A mutant is catalytically intact *in vitro* (Thuille et al., 2005). Similarly, PKC-θ T219A mutant shows slightly increased lipid-binding activity with PDBu (Thuille et al., 2005). These data suggest that T219 phosphorylation regulates PKC-θ-mediated downstream signaling in T cells through a mechanism independent of PKC-θ kinase activity (Thuille et al., 2005). In contrast, PKC-θ T219A mutant is unable to translocate into lipid rafts or the immunological synapse in Jurkat T cells in response to TCR stimulation or in the presence of antigen-presenting cells; forced membrane translocation of PKC-θ T219A mutant by adding a membrane-targeting sequence reconstitutes TCR-induced NF-κB activation in Jurkat T cells (Thuille et al., 2005). Taken together, these data suggest that T219 phosphorylation is required for properly localizing PKC-θ to the cell membrane, allowing PKC-θ to activate downstream effectors in TCR signaling.

### Phosphorylation of PKC-θ At T538 in the Activation Motif

#### Identification of PKC-θ autophosphorylation sites at the catalytic domain

Multiple sequence alignment of the catalytic domains of PKC isoforms shows the conserved serine/threonine phosphorylation residues in the activation loop, turn motif, and hydrophobic motif (Liu et al., 2002; Xu et al., 2004). Phosphorylations of T538 at the activation loop, S676 at the turn motif, and S695 at the hydrophobic motif of PKC-θ have been demonstrated using phospho-specific anti-sera and further confirmed by mass spectrometry (Liu et al., 2002; Czerwinski et al., 2005). Phosphorylation of S685 at the turn motif has been demonstrated only by mass spectrometry (Czerwinski et al., 2005). T538, S676, and S695 are constitutively phosphorylated on recombinant PKC-θ isolated from baculovirus, *E. coli*, or HEK293T expression systems (Liu et al., 2002; Czerwinski et al., 2005; Thuille et al., 2005). The PKC-θ kinase-dead mutant (K409W) abolishes phosphorylation of these three sites in the recombinant PKC-θ, suggesting that they are regulated by autophosphorylation (Liu et al., 2002; Czerwinski et al., 2005). Autophosphorylation can be induced in a *cis* or *trans* manner. An intrapeptide reaction in a mixed micelle system shows that PKC-θ autophosphorylation is regulated by intrapeptide phosphorylation (Newton and Koshland, 1987). Catalytic-competent PKC-θ is unable to phosphorylate PKC-θ-derived peptides containing the individual autophosphorylation sites, further supporting that an intramolecular interaction is required for autophosphorylation on these sites (Thuille et al., 2005). Crystal structure of PKC-θ shows that the activation loop, the turn motif, and hydrophobic motif lie close to the active cleft (Xu et al., 2004), supporting that T538, S676, and S695 are autophosphorylated in *cis*. So far, the function and regulation of T538 phosphorylation are the best known and the most intensively studied, whereas the functions and regulations of other three phosphorylation sites in T cells are less clear.

#### Function of PKC-θ T538 phosphorylation

The activation loop is a short critical polypeptide that lies outside the active site cleft of the kinase domain (Nolen et al., 2004). The activation loop contributes to binding surfaces for substrates and co-factors (Nolen et al., 2004). The crystal structure of PKC-θ shows that the phosphate of T538 in the activation loop forms hydrogen-bonding interaction with the αC helix, which helps to stabilize the correct orientation of the αC helix and to maintain active conformation of this kinase (Xu et al., 2004). Loss of T538 phosphorylation in PKC-θ T538A mutant abolishes PKC-θ kinase activity, indicating that T538 is a critical activation site regulating PKC-θ kinase activity (Liu et al., 2002; Czerwinski et al., 2005). Therefore, T538 phosphorylation is widely used as a surrogate marker for PKC-θ kinase activation. T538 phosphorylation does not seem to regulate PKC-θ membrane translocation, because PKC-θ T538A mutant still translocates into lipid rafts in T cells upon anti-CD3/CD28 stimulation (Thuille et al., 2005). Consistent with loss of kinase activation of PKC-θ T538A mutant, the downstream NF-κB and NF-AT activations are abrogated in PKC-θ T538A-transfected Jurkat T cells upon anti-CD3/CD28 costimulation (Liu et al., 2002; Thuille et al., 2005), indicating that T538 is a critical phosphorylation site required for PKC-θ function and T cell activation.

#### Inducible PKC-θ T538 phosphorylation in T cell signaling

While it is undoubtedly true that T538 is constitutively autophosphorylated in the recombinant PKC-θ proteins isolated from different expression systems (Liu et al., 2002; Xu et al., 2004; Czerwinski et al., 2005; Thuille et al., 2005), whether PKC-θ T538 phosphorylation is constitutive or inducible during T cell activation has been controversial. A constitutive T538 phosphorylation of endogenous PKC-θ has been detected in Jurkat and primary T cells using the phospho-specific antibodies from different sources, and T538 phosphorylation is not further enhanced by TCR or PMA stimulation (Freeley et al., 2005; Thuille et al., 2005; Gruber et al., 2006). The observation of constitutive PKC-θ T538 phosphorylation in T cells reported in some studies maybe due to high basal PKC-θ activity; for example, Jurkat T cells can be easily stimulated with serum (Lee et al., 2006). Earlier studies of PKC-θ phosphorylation in T cells used an electrophoretic mobility shift of PKC-θ in T cells upon anti-CD3/CD28 or PMA/ionomycin stimulation; these studies show that PKC-θ migrates more slowly in activated T cells than in unstimulated T cells (Thebault and Ochoa-Garay, 2004; Puente et al., 2005). Although the phosphorylation of PKC-θ at T538 is not directly examined in these studies due to the lack of use of anti-phospho-PKC-θ (T538) antibody, PKC-θ T538A mutation abolishes the mobility shift of PKC-θ induced by PMA (Thebault and Ochoa-Garay, 2004; Puente et al., 2005), suggesting that T538 phosphorylation is induced by T cell signaling. Using the phospho-specific PKC-θ T538 antibodies from different sources, several groups show that T538 phosphorylation is induced during T cell activation upon anti-CD3 or interferon stimulation (Srivastava et al., 2004; Lee et al., 2005; Liu and Lai, 2005; Park et al., 2009; Chuang et al., 2011). The inducible T538 phosphorylation of PKC-θ is consistent with the inducible PKC-θ kinase activation in T cells upon TCR stimulation (Monks et al., 1997; Coudronniere et al., 2000; Puente et al., 2005). Furthermore, our group has identified GLK as the direct upstream kinase that phosphorylates PKC-θ T538 upon TCR stimulation (Chuang et al., 2011). Therefore, PKC-θ-dependent downstream signaling such as IKK-NF-κB activation may need to be examined as controls for the basal PKC-θ activity in unstimulated T cells (Lee et al., 2005,^2006^). Taken together, PKC-θ T538 phosphorylation and its kinase activation are induced in T cells upon TCR stimulation.

#### Regulation of PKC-θ T538 phosphorylation and activation by GLK in TCR signaling

The kinase that directly phosphorylates PKC-θ was a mystery before the discovery of the role of GLK in TCR signaling. Initially, 3-phosphoinositide-dependent kinase-1 (PDK1) was proposed to be the kinase directly phosphorylating PKC-θ based on the following evidence: (i) PKC-θ T538 phosphorylation is impaired in PDK1-knockdown Jurkat T cells and PDK1-deficient primary T cells upon anti-CD3/CD28 stimulation (Lee et al., 2005; Park et al., 2009); (ii) PDK1 is associated with PKC-θ in the lipid rafts of T cells during anti-CD3/CD28 stimulation (Lee et al., 2005); (iii) PDK1 directly phosphorylates the conserved phosphorylation site at the activation loop of the other PKC isoforms (Le Good et al., 1998; Balendran et al., 2000). However, several observations indicate that PDK1 may not be the direct upstream kinase for PKC-θ: (i) anti-CD3 stimulation alone without anti-CD28 costimulation is sufficient to induce phosphorylation and activation of PKC-θ but not PDK1 (Liu and Lai, 2005; Park et al., 2009); (ii) PDK1-deficient T cells still show residual PKC-θ T538 phosphorylation (Park et al., 2009); and (iii) there is lack of *in vitro* phosphorylation of PKC-θ by PDK1 to demonstrate that PKC-θ is a direct substrate of PDK1. A recent study suggests that AMP-activated protein kinase (AMPK) may be involved in PKC-θ T538 phosphorylation in Jurkat T cells upon PMA/ionomycin stimulation (Lee et al., 2012). In addition, APMK is also implicated in phosphorylation of PKC-ζ on its activation loop in the cells under the hypoxia (Gusarova et al., 2009). However, there is no direct evidence that AMPK directly phosphorylates PKC-θ at T538 in TCR signaling (Lee et al., 2012).

GLK is a Ste20-like serine/threonine kinase that activates the JNK pathway in response to stress stimulation (Diener et al., 1997). Our recent study of the GLK function in T cells revealed an important role of GLK in controlling TCR signaling, T cell activation, immune responses, and autoimmunity via activating PKC-θ (Chuang et al., 2011). GLK-deficient T cells show abolished phosphorylation of PKC-θ T538 and IKK, as well as reduced activation of NF-κB, upon anti-CD3 stimulation; this effect is unlikely mediated by PDK1 because PDK1 is not activated by anti-CD3 stimulation alone (Park et al., 2009; Chuang et al., 2011). Furthermore, PDK1 activation is unaffected in GLK-deficient T cell upon anti-CD3/CD28 costimulation, even though phosphorylations of PKC-θ and IKK are abolished in these cells (Chuang et al., 2011). These data indicate that GLK activates PKC-θ independent or downstream of PDK1. GLK is inducibly and directly associated with PKC-θ in T cells upon anti-CD3 stimulation (Chuang et al., 2011). GLK directly phosphorylates PKC-θ at T538 but not at S695 or S676 residue *in vitro* (Chuang et al., 2011). These data unequivocally demonstrate that GLK is the kinase that directly phosphorylates and activates PKC-θ during TCR signaling. Our unpublished data show that PKC-θ is unable to translocate to cell membrane in GLK-deficient T cells upon anti-CD3 stimulation. This effect is not likely mediated by a loss of PKC-θ T538 phosphorylation in GLK-deficient T cells, because T538A mutation does not affect PKC-θ membrane translocation (Thuille et al., 2005). How GLK regulates PKC-θ membrane translocation remains unknown. One potential mechanism is that GLK may directly or indirectly induce another phosphorylation site (e.g., T219 or a novel S/T residue) that regulates PKC-θ membrane translocation. Further characterization of GLK-mediated PKC-θ phosphorylation may reveal a novel mechanism that regulates PKC-θ membrane translocation.

### PHOSPHORYLATION OF PKC-θ AT S676 AND S685 IN THE TURN MOTIF

S676 is constitutively autophosphorylated in the turn motif of recombinant PKC-θ isolated from HEK293T cells and *E. coli* expression system (Liu et al., 2002; Czerwinski et al., 2005). Basal S676 phosphorylation of PKC-θ has also been observed in Jurkat T cells, CTL clone AB.1 cells, and primary CD4^+^ T cells (Freeley et al., 2005; Puente et al., 2005; Lee et al., 2010). The basal level of PKC-θ S676 phosphorylation is moderately increased in mouse primary CD4^+^ T cells upon anti-CD3/CD28 costimulation (Lee et al., 2010). However, some controversy remains about the effects of S676 phosphorylation on the PKC-θ kinase activity and downstream NF-κB activation (Liu et al., 2002; Czerwinski et al., 2005; Thuille et al., 2005). PKC-θ S676A mutant isolated from Jurkat T cells or *E. coli* expression system retains only 30–40% *in vitro* kinase activity compared to WT (Czerwinski et al., 2005; Thuille et al., 2005); furthermore, PKC-θ S676A mutant dramatically suppresses NF-κB activity in Jurkat T cells stimulated by phorbol ester or anti-CD3 (Thuille et al., 2005). In contrast, PKC-θ S676A mutant isolated from HEK293T cells displays intact kinase activity and does not affect TCR-induced NF-κB activity in Jurkat T cells (Liu et al., 2002). These different results could be due to using experimental systems or conditions. It has been observed in PKC-βII that a single mutation of a conserved serine/threonine residue in the turn motif does not affect PKC-βII function due to compensatory phosphorylation at the neighboring residues (Edwards et al., 1999). In a similar note, combinational mutations of S676 and other neighboring serine residues (S662 and S685) in the turn motif of PKC-θ result in a greater loss of its kinase activity (Liu et al., 2002).

Mass spectrometry studies using PKC-θ isolated from *E. coli* expression system reveal that S685 is an autophosphorylation site in the turn motif of PKC-θ (Czerwinski et al., 2005). Another mass spectrometry study using PKC-θ isolated from Jurkat T cells shows that S685 phosphorylation is induced in Jurkat T cells upon anti-CD3 stimulation (Mayya et al., 2009). Although S685 phosphorylation of PKC-θ remains to be further verified using phospho-specific antibody, PKC-θ S685A mutant shows significantly reduced kinase activity (Czerwinski et al., 2005). The effect of S685 phosphorylation on the downstream NF-κB activation has not been reported; but it would be expected to positively regulate PKC-θ function and T cell activation during TCR signaling. How the phosphorylation at the turn motif affects PKC-θ catalytic activity is not clear. The electron density maps in crystallization studies of PKC-θ do not define the turn motif (Xu et al., 2004); however, the crystallization studies of the kinase domains of PKC-βII and PKC-ι show that the phosphate on the turn motif forms intramolecular ionic contacts to help stabilize the active conformation of these enzymes (Messerschmidt et al., 2005; Grodsky et al., 2006). Therefore, the phosphorylation at the turn motif of PKC-θ may contribute to the regulation of its catalytic activity.

### PHOSPHORYLATION OF PKC-θ AT S695 IN THE HYDROPHOBIC MOTIF

S695 is a constitutive autophosphorylation site in the C-terminal hydrophobic motif of PKC-θ when isolated from *E. coli*/baculovirus/HEK293T expression systems (Liu et al., 2002; Xu et al., 2004; Czerwinski et al., 2005; Thuille et al., 2005). In Jurkat T cells, human primary T cells, and mouse primary T cells, S695 phosphorylation of PKC-θ is induced upon anti-CD3 with or without CD28 costimulation (Villalba et al., 2002; Freeley et al., 2005; Lee et al., 2010). A deficiency of an essential mTOR complex 2 (mTORC2) subunit RICTOR suppresses PKC-θ phosphorylation at S696 but not at S676 in mouse primary CD4^+^ T cells upon anti-CD3/CD28 stimulation. Thus, mTORC2 regulates S696 phosphorylation of PKC-θ during TCR signaling. S696 phosphorylation may contribute to catalytic activity of PKC-θ, which is important for mTORC2-mediated Th2 differentiation (Lee et al., 2010). PKC-θ S695A mutant diminishes PKC-θ *in vitro* kinase activity, supporting that S695 phosphorylation is required for optimal PKC-θ kinase activation (Liu et al., 2002; Czerwinski et al., 2005; Thuille et al., 2005). Crystallographic data of PKC-θ catalytic domain further support that S695 phosphorylation contributes to an active conformation of PKC-θ by tightening intramolecular interaction between the hydrophobic motif and the N-lobe to align the αC helix (Xu et al., 2004). Phosphorylation of PKC-βII at the hydrophobic motif regulates subcellular localization in addition to catalytic activity of this kinase (Edwards et al., 1999). Membrane-associated PKC-θ, but not cytosolic PKC-θ, is phosphorylated at S695, raising the possibility that S695 phosphorylation may regulate membrane translocation of PKC-θ (Villalba et al., 2002; Freeley et al., 2005). Two lines of evidence suggest that S695 phosphorylation may not be involved in membrane translocation of PKC-θ: (i) the conventional PKC inhibitor Gö6976 abrogates S695 phosphorylation without affecting membrane translocation of PKC-θ in Jurkat T cells upon anti-CD3/CD28 stimulation (Freeley et al., 2005); and (ii) the N-terminal regulatory region but not the C-terminal kinase domain regulates membrane translocation of PKC-θ during T cell activation (Bi et al., 2001; Thuille et al., 2005). Future studies of PKC-θ membrane translocation in T cells using S695A mutant will provide a conclusive answer. The effect of S695 phosphorylation of PKC-θ on T cell activation varies in different studies. Catalytically active PKC-θ A148E mutant induces NF-κB and NF-AT activation in Jurkat T cells upon anti-CD3/CD28 costimulation, which is greatly inhibited by S695A mutation in PKC-θ A148E mutant (Thuille et al., 2005). In contrast, another study shows that PKC-θ S695A mutant does not affect NF-κB activation in the Jurkat T cells upon anti-CD3/CD28 costimulation; paradoxically, the kinase activity of PKC-θ S695A mutant is impaired in the same study (Liu et al., 2002). However, the regulation of PKC-θ kinase activation by S695 phosphorylation supports that S695 phosphorylation of PKC-θ positively regulates T cell activation during TCR signaling.

#### Interdependency of different PKC-θ autophosphorylation sites

Previous studies suggest interdependency of different PKC-θ autophosphorylation sites. Loss of T538 phosphorylation in PKC-θ T538A mutant leads to reduced or abolished S676 and S695 phosphorylation in HEK293 cells (Liu et al., 2002). This is likely the consequence of low catalytic activity of T538A mutant, since S676 and S695 are autophosphorylated on the recombinant PKC-θ protein *in vitro* (Liu et al., 2002). In addition, treatment with a serine/threonine phosphatase inhibitor okadaic acid restores the S676 and S695 phosphorylation but not the catalytic activity of PKC-θ T538A mutant (Liu et al., 2002), supporting an additional or alternative explanation that mutation of one phosphorylation site may affect the phosphorylation status at another site by increasing the susceptibility of this mutant to dephosphorylation by phosphatases. On the other hand, PKC-θ S695A mutant results in a great loss of T538 phosphorylation in HEK293 cells and reduced *in vitro* kinase activity (Liu et al., 2000; Czerwinski et al., 2005); this effect may be attributed to a secondary effect of reduced catalytic activity of S695A mutant. However, this does not seem to apply to PKC-θ S676A mutant, which shows reduced catalytic activity but appears to have intact T538 phosphorylation in HEK293 cells (Liu et al., 2002; Czerwinski et al., 2005). The unphosphorylated turn motif in PKC-βII is bound to HSP70, which stabilizes PKC-βII, allowing re-phosphorylation of this enzyme (Gao and Newton, 2002); this study suggests that PKC-θ S676A may be more stable and ready for autophosphorylation at T538 due to a loss of phosphorylation in the turn motif of this mutant. Another possibility is that PKC-θ T538 can be phosphorylated by other kinases. The relationship of S676 and S695 phosphorylation has not been reported. Phosphorylations of T219 and T538 in PKC-θ are independent of each other, as mutation of either site does not affect phosphorylation on the other site on baculovirus-expressed PKC-θ proteins (Thuille et al., 2005). This finding supports that T219 phosphorylation does not regulate the catalytic activity of PKC-θ (Thuille et al., 2005); however, it is not clear why T219 autophosphorylation is not affected by T538A mutation. One possibility is that T219 of PKC-θ could also be regulated by other kinases. While multiple residues of PKC-θ are constitutively autophosphorylated *in vitro*, the relationship of these phosphorylation residues during TCR signaling has not been studied. Is there sequential phosphorylation of different sites on PKC-θ upon TCR stimulation? Is there spatial regulation of PKC-θ phosphorylation during T cell activation? Future studies of these questions may reveal a novel regulation of PKC-θ activation and function by phosphorylation.

## CONCLUDING REMARKS

**FIGURE 3:**
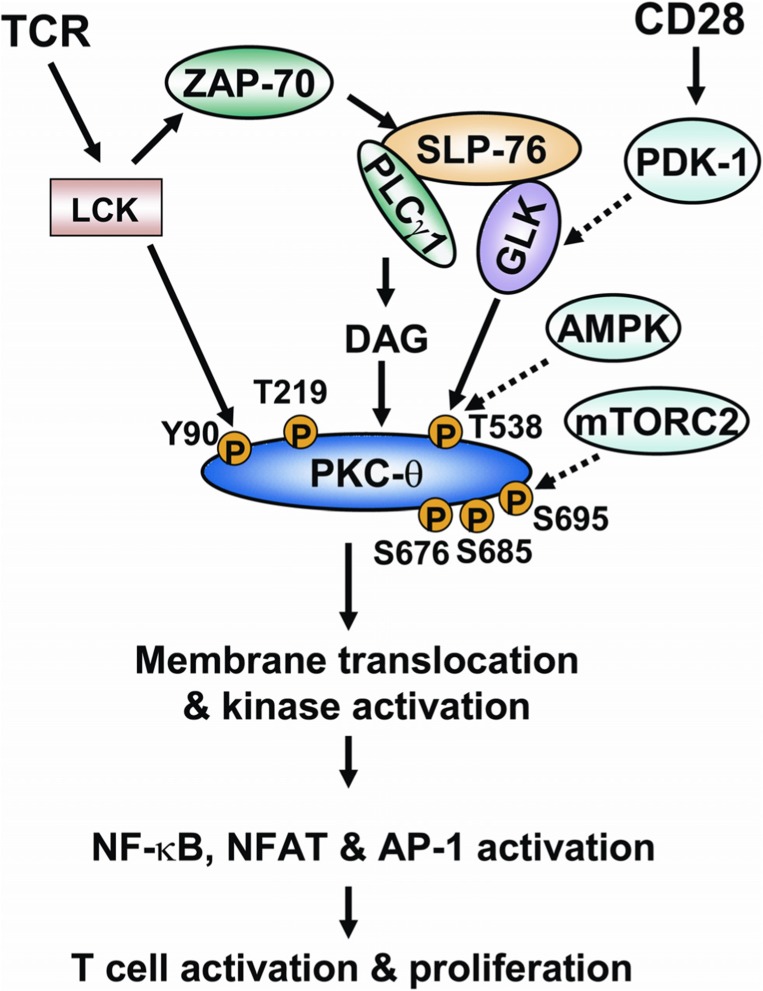
**The model of regulation of PKC-θ phosphorylation during TCR signaling.** Ligation of TCR induces the activation of tyrosine kinase LCK. LCK activates ZAP-70, which in turn induces tyrosine phosphorylation of the adaptor SLP-76. SLP-76 directly interacts with and activates PLCγ1 and GLK. PLCγ1 cleaves a phospholipid, generating the second messenger diacylglycerol. The binding of diacylglycerol with PKC-θ induces the conformational change of PKC-θ; T538 of PKC-θ is then phosphorylated by GLK, leading to catalytic activation of PKC-θ. The CD28 costimulatory activates PDK-1, which facilitates PKC-θ T538 phosphorylation possibly via activating GLK. AMPK is also implicated in PKC-θ T538 phosphorylation in T cells. LCK directly interacts with and phosphorylates PKC-θ at Y90. The phosphorylation of Y90 and another residue (T219) induces membrane translocation of PKC-θ. S676, S685, and S695 residues are also phosphorylated on PKC-θ upon TCR stimulation. mTORC2 activity is required for PKC-θ S695 phosphorylation in T cells. Catalytic activation and membrane translocation of PKC-θ lead to the activation of transcription factors NF-κB, NF-AT, and AP-1, and to subsequent T cell activation.

In conclusion, phosphorylations play critical roles in regulating PKC-θ catalytic activity and membrane translocation, both of which are required for the proper function of PKC-θ in T cell activation. The regulation of PKC-θ phosphorylation is much more complex than we initially thought. The individual PKC-θ phosphorylation sites can be induced by autophosphorylation or upstream kinases depending on different cell types or cell stimuli. In TCR signaling, T538 and Y90 are phosphorylated on PKC-θ by GLK and LCK, respectively. Although phosphorylations of T219, S676, S685, and S695 are all induced during TCR signaling, the direct kinases for the phosphorylation of these sites on PKC-θ remain unclear. T538 phosphorylation is most critical for PKC-θ catalytic activation, whereas S676, S685, and S695 may be required for optimal catalytic activation of PKC-θ during TCR signaling. Phosphorylations of Y90 and T219 regulate PKC-θ membrane translocation and T-cell activation. PKC-θ is recruited to different locations in effector T cells and regulatory T cells and performs opposite functions in these cells (Zanin-Zhorov et al., 2010). For example, PKC-θ localizes to the immunological synapse and positively regulates cellular activation in effector T cells, while PKC-θ is sequestered away from the immunological synapse in stimulated regulatory T cells and negatively regulates cell function (Zanin-Zhorov et al., 2010). On a similar note, a substantial amount of PKC-θ is constitutively located in the nucleus of T cells, where it regulates gene expression (Sutcliffe et al., 2011). It might therefore be appropriate to speculate that different phosphorylation sites (e.g., Y90, T219, or other sites) of PKC-θ may influence its distribution to different subcellular locations. The model of regulation of PKC-θ phosphorylation in TCR signaling is shown (**Figure 3**). How such a complex regulation of PKC-θ phosphorylation is integrated with TCR/CD28 signaling is the key for the future understanding of PKC-θ function and T cell activation. Specifically, what kinases or phosphatases regulate the important PKC-θ phosphorylation sites in T-cell signaling? Does the phosphorylation of PKC-θ at different residues occur in a sequential and spatial manner during T cell signaling? How does PKC-θ phosphorylation at a specific site regulate its catalytic activity and membrane translocation? Future studies of the regulatory mechanisms of PKC-θ phosphorylation are important not only for the understanding of the underlying mechanism of PKC-θ-mediated cell activation, but also for the design of novel therapeutic targets for PKC-θ-mediated inflammatory diseases.

## Conflict of Interest Statement

The authors declare that the research was conducted in the absence of any commercial or financial relationships that could be construed as a potential conflict of interest.
